# The influence of the largest private shareholder on bank loans: Evidence from China

**DOI:** 10.1371/journal.pone.0276877

**Published:** 2022-10-27

**Authors:** Jie Liu, Limei Xu, Qiaoyun Zhang

**Affiliations:** 1 School of Finance, Institute of Chinese Financial Studies, Southwestern University of Finance and Economics, Chengdu, Sichuan, China; 2 Chengdu High-tech Industrial Development Zone Sub-branch, Bank of China, Chengdu, Sichuan, China.; University of Almeria: Universidad de Almeria, SPAIN

## Abstract

We point out that the largest private shareholders can use their information advantage of the industry to influence banks’ industry lending behavior. Using a sample of Chinese city commercial banks, we find that increasing of ownership stake of the largest private shareholders leads banks to lend more to their industries. Interestingly, the largest state-owned shareholders do not have this effect. More importantly, we confirmed the channel of the information advantage by analyzing the bank’s industry NPL ratio and the listed company’s maximum loan amount in the bank. Of course, the effect of the largest private shareholders is achieved by intervening in board decisions. In addition, the ownership structure can influence this effect.

## Introduction

The largest private shareholder has a significant impact on bank operations. However, current studies have focused more on the influence of state-owned shareholders on bank lending behavior, because government shareholding is a common phenomenon in many countries [1]. It should be pointed out that the ownership stake of private shareholder in banks is an important manifestation of the reform of the ownership structure of China’s banking industry. Since the reform, taking Chinese city commercial banks as an example, almost all banks are partially owned by private shareholder. The role of private shareholders should receive more attention. The core of the financial system reform is to improve the efficiency of financial resource allocation, and rationally optimizing the industrial allocation of bank loans is the key. Generally, the influence of large shareholders is more important. Therefore, we study the following issues based on the industry background of the largest private shareholders. What is the effect of the largest private shareholder on the industry preference of bank loans? What is the channel through which the largest private shareholders influence the industry choice of bank loans? These issues are crucial. Studying these issues not only enriches and perfects the corporate governance and management theories of China’s banking industry. And in practice, it helps to guide private enterprises to participate in the industry allocation of credit, thereby improving the efficiency of resource allocation and promoting the upgrading of economic structure. Our research on these issues is very essential for the regulatory discussions and financial stability especially in a developing country setting like China.

**Fig 1:**
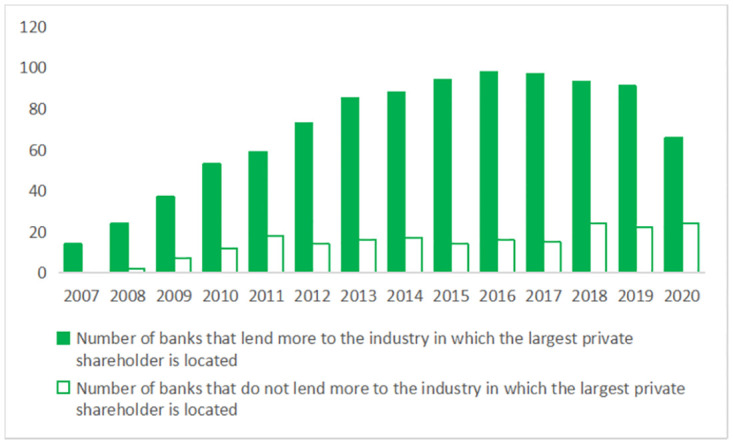
Number of banks that lend more to the industry in which the largest private shareholder is located. The figure plots the number of banks that lend more to the industry in which the largest private shareholder is located (solid bars) vs. the number of banks that do not lend more to the industry (hollow bars) for each year. Based on the proportion of banks’ loans to various industries, we define that banks have made more loans to the top five industries, otherwise, they have not.

**Fig 2:**
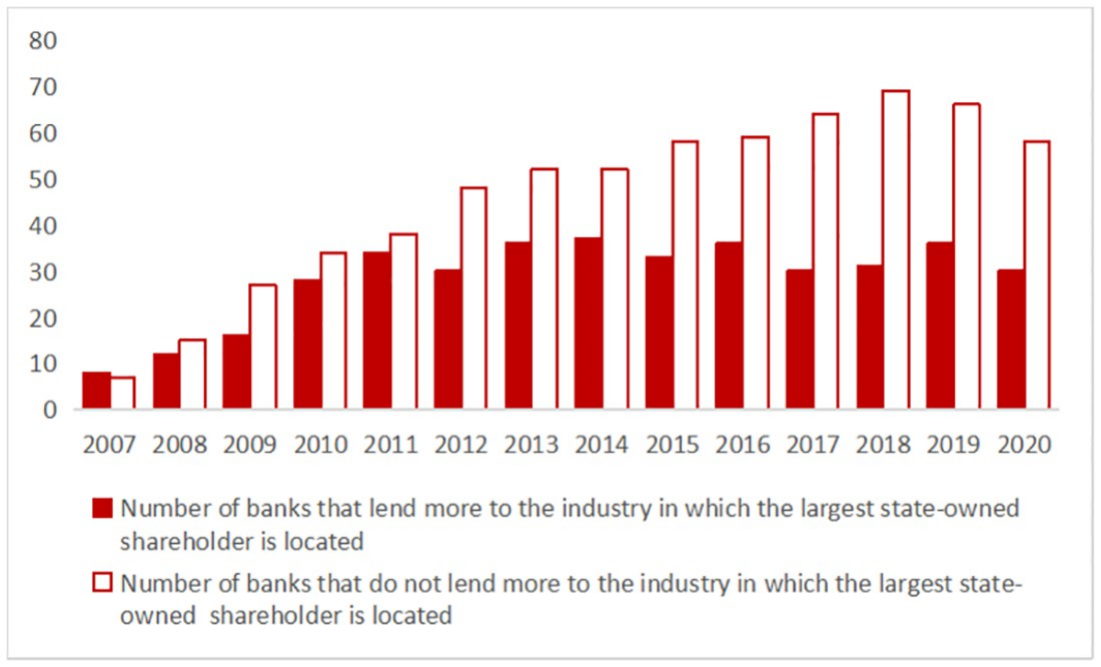
Number of banks that lend more to the industry in which the largest state-owned shareholder is located. The figure plots the number of banks that lend more to the industry in which the largest state-owned shareholder is located (solid bars) vs. the number of banks that do not lend more to the industry (hollow bars) for each year. Based on the proportion of banks’ loans to various industries, we define that banks have made more loans to the top five industries, otherwise, they have not.

The theory of information asymmetry suggests that if banks lack information about a certain industry, banks may reduce or deny lending to that industry. Enterprises often have a clearer information advantage in their industry. Therefore, the industry information held by bank shareholders may influence the industry choice of loans. At the same time, state-owned shareholders of Chinese banks have to consider more political factors [2–5]. The difference is that the influence of private shareholders on industry choices of loans is more based on market factors, their own preferences and interests [6], so the information advantages can play a special role in bank loans. As the largest private shareholder’s ownership stake in the bank increases, thier information advantage is particularly valuable and can be exerted. This makes it cheaper for banks to acquire information about the industry in which the largest private shareholder is located, thereby facilitating lending to the industry. Indeed, as Fig 1 shows, there are significantly more banks that lend more to industries in which the largest private shareholders are located than those that do not. In contrast, we find in Fig 2 that the number of banks that made more loans to the industry of the largest state-owned shareholder is less than the number of banks that do not.

Although banks have provided more loans to the industries where the largest private shareholders are located. However, to accurately identify the influence of the largest private shareholder on the industry choice of bank loans, rigorous empirical analysis is required. Using a bank-level dataset of Chinese city commercial banks from 2007 to 2020, we find that as the ownership stake of the largest private shareholder increases, banks increase their lending to the industry of the largest private shareholder. As a comparison, we also empirically test the influence of the largest state-owned shareholder on bank loans, and find that there is no similar influence. This coincides with some of the findings of Clarke et al. [7], Berkowitz et al. [8], Shaban and James [9] and Yuan et al. [10]. Our study also shows that the largest private shareholder has a different influence than the state-owned shareholder.

Another important issue we need to understand is what channel does the largest private shareholder use to influence bank lending behavior? We believe this is due to the information advantage of the largest private shareholders on their industry. We conducted channel tests from two aspects, one is to analyze the influence of the largest private shareholder on the NPL ratio of the bank’s industry loans, and the other is to verify whether the firms in the same industry are to obtain this bank more loans. We find that when the largest private shareholder has more ownership stake, banks have lower NPL ratios for loans to the industry in which the largest private shareholder is located. Also, banks have higher maximum loan amount to the firms in the same industry. Our empirical research shows that this channel of the influence of the largest private shareholders on the bank loans is that they have an information advantage in the industry.

To ensure the rigour of the study, we performed robustness checks from multiple aspects. One is to solve the endogeneity between the largest private shareholder’s ownership stake in the bank and the bank’s loan behavior. We use the average value of the largest private shareholder’s ownership stake of other banks located in the same region in the initial year of the bank sample (excluding their own situation) as an instrumental variable, and perform 2SLS regression. Second, in order to exclude the factor that the industry where the largest private shareholder located accounts for a large share in the overall economy, which leads to the high proportion of bank loans to this industry, we deleted the samples of industries that ranked high in the regional economy. The third is to conduct a group test according to the size of the bank’s assets to avoid the influence of the bank’s own characteristics. The fourth is to confirm that the influence of the largest private shareholder on bank loans is different from that of state-owned shareholders. We test that the ownership stake of the largest state-owned shareholder have no influence on the industry choice of bank loans.

Additionally, we find that the largest private shareholder influences banks’ lending behavior by interfering in the board decisions, rather than by acting as a bank manager. The ownership structure of a bank can significantly affect the effect of the largest private shareholder. Specifically, the higher the ownership structure concentration and the higher the ownership stakes of the largest state-owned shareholder, the weaker the effect of the largest private shareholder. The higher the degree of dispersion (diversification) of the ownership structure, the greater the effect of the largest private shareholder.

Our research perspective has great innovation. The impact of ownership structure on bank lending behavior has been extensively studied, but these are more based on the perspective of state ownership [11–14]. Private shareholders have already played an important role in city commercial banks, so it is necessary to study their influence on bank lending behavior from the perspective of private shareholders. Furthermore, we not only focus on the ownership stake of the largest private shareholders, but also emphasize the influence of their industry background, we provide a new perspective for studying the shareholding structure on bank lending behavior.

Our research content also has great innovation. There are more literatures on bank lending behavior from the aspects of loan amount, loan interest rate and loan concentration [2, 15, 16]. The difference is that we study bank lending behavior based on the industry distribution of bank loans. This can not only describe the allocation of loan resources more specifically and accurately, but also enrich the content of studying bank lending behavior.

Channel is a prominent contribution of our research. This is different from simply analyzing the relationship between shareholders and bank lending behavior. Our research also shows that the information advantage works only if shareholders themselves are critical in corporate governance. The largest private shareholders (with higher ownership stakes and less government intervention) can use their information advantage to influence bank lending. Of course, the way to influence is by intervening in board decisions.

The significance of our study is critical. On the one hand, our conclusions help to deeply understand the role of the largest private shareholder on bank operations. On the other hand, it can guide the allocation of bank loans, so that financial resources can match the national industrial development strategy. In addition, it has certain reference significance for the introduction of relevant supervision and regulation policies. Regulatory policies should fully recognize the influence of the industry background and ownership stake of the largest private shareholder on bank lending behavior.

## Theoretical analysis and hypothesis

We point out that the information advantage in the industry of the largest private shareholders is the channel through which they influence the industry choice of bank loans. Market-oriented lending behavior requires banks to make a reasonable assessment of investment projects based on borrowers’ information. Generally, banks are information disadvantaged. Banks can only obtain public financial information of borrowers, but it is difficult to obtain private information such as corporate culture, human capital, etc. [17]. The banks’ degree of information mastery for the borrower is the basic factor affecting the lending behavior [18]. If banks cannot effectively obtain and screen information of borrowers, banks may decide to lend less or not even lend to such borrowers.

The most straightforward way to resolve information asymmetry is to send information to counterparties [19]. The largest private shareholders can provide relevant information about their industry, including special and critical information such as corporate culture, human capital, etc. Therefore, the largest private shareholder can help banks determine who is the “bad” borrower in the industry, thereby avoiding “adverse selection”. In addition, this information advantage enables banks to avoid “moral hazard” by formulating debt contracts that constrain the behavior of borrowers in the industry after obtaining loans.

The existence of the largest private shareholder can alleviate the information asymmetry suffered by banks. Because the information held by the largest private shareholders about the industry is often proprietary, it can yield insights into the industry not available from other sources. Such insights can reduce the bank’s cost of collecting information of borrowers in the same industry, thereby reducing the NPL ratio and facilitating these firms in the industry to obtain more loans. Therefore, through the channel of information advantage, we realize that it is a rational behavior for banks to issue more loans to the industry where the largest private shareholder is located.

Note that this information advantage is relative. That doesn’t mean they have all the information about companies in their industry. But, they are definitely more professional and convenient than laymen for information acquisition. In addition, whether shareholders can play the role of information advantage, and then affect bank lending behavior depends on their own characteristics. Shareholders need larger ownership stakes and are independent (free from government interference). Therefore, this advantage is difficult to exert effectively for smaller shareholders (whose role is restricted by major shareholders) and the largest state-owned shareholder (subject to the government intervention). Here, we make the **Hypothesis 1** and the **Hypothesis 2**:

**Hypothesis 1.** The higher the largest private shareholder’s ownership stake in the bank, the higher the proportion of the bank’s loan to the industry where the largest private shareholder is located.
**Hypothesis 2.** The largest private shareholder’s influence channel on bank lending behavior is their information advantage about the industry.

## Data and methods

Based on data availability, we use 126 city commercial banks in China, and the sample is the unbalanced panel data from 2007 to 2020. The shareholding structure data, industry loan data and other financial data of the sample banks are partly derived from the Wind database. In the field of financial data, Wind database has built a complete and accurate large-scale financial database with financial securities data as the core in China. The content of the Wind database covers stocks, commercial banks, bonds, funds, foreign exchange, financial derivatives, macroeconomics, and other fields. Data for non-listed banks is manually compiled according to the annual reports published on the official websites of each bank. Macroeconomic data measuring regional economic characteristics were obtained from the Wind database and the National Bureau of Statistics of China. The loan data of listed firms is obtained from the CSMAR (China Stock Market & Accounting Research Database) database. The CSMAR database records in detail the loan amount of various banks to listed firms, including the basic information and financial data of listed firms and the maximum loan amount approved by banks to firms.

We measure the power of the largest private shareholder by *LPS*, their ownership stake in the bank. We use *IndLoan*, the ratio of loans to the industry of the largest private shareholder to bank’s total loans, to measure the industry choice of bank lending. As shown in Panal A of Table 1, the mean of *IndLoan* is 0.158. That is banks’ loans to the industry of the largest private shareholder accounted for 15.8% of the total annual loans on average, *IndLoan* has a standard deviation of 13.1% and can swing up to 78.2%. The mean of *LPS* is 0.078, indicating that the largest private shareholder owns 7.8% of the bank’s ownership stake on average, with a maximum of 50%.

**Table 1:** Summary statistics. This table reports the summary statistics. Regression variables are defined in S1 Appendix. The sample period is from 2007 to 2020.

Panal A: Main variables					
Variables	mean	sd	min	max	N
*IndLoan*	0.158	0.131	0	0.782	1216
*LPS*	0.078	0.052	0	0.5	1216
*IndNPL*	0.022	0.03	0	0.251	165
*FirmLoan*(100 mln CNY)	2.205	3.592	0	20	735
*Ln*(1+ *FirmLoan*)	0.832	0.723	0	3.045	735
*IndLoanS*	0.059	0.089	0	0.821	1125
*LSS*	0.114	0.136	0	0.87	1125
Panal B: Characteristic variables at the bank level					
Variables	mean	sd	min	max	N
*Stat*	0.044	0.206	0	1	1216
*Priv*	0.035	0.183	0	1	1125
*Size* (CNY)	25.43	1.089	22.4	28.7	1216
*LDR*	0.626	0.126	0.167	1.577	1216
*Fore*	0.294	0.456	0	1	1216
*CAR*	0.135	0.036	0.056	0.596	1216
Panal C: Characteristic variables of regional macroeconomics					
Variables	mean	sd	min	max	N
*GDPr*	0.083	0.032	-0.025	0.174	1216
*Deptr*	0.125	0.059	-0.05	0.4	1216
*SOE*	0.323	0.317	0.055	2.912	1216
*GDP*_*sec*	0.457	0.068	0.162	0.615	1216
*GDP*_*thir*	0.454	0.077	0.286	0.835	1216
Panal D: Characteristic variables at the firm level					
Variables	mean	sd	min	max	N
*Peer*	0.174	0.379	0	1	735
*LnAssets* (CNY)	23	1.396	18.83	26.67	735
*Leverage*	0.565	0.189	0.063	1.175	735
*Current*	0.559	0.218	0.057	0.992	735
*Profitability*	0.013	0.118	-1.47	0.323	735
*Capx*	0.041	0.047	0	0.372	735

In addition to testing the effect of the largest private shareholder on the industry choice of bank lending, we also examine the channel. We use *IndNPL*, the NPL ratio of bank’s loans to the industry of the largest private shareholder, and *FirmLoan*, the maximum loan amount approved by banks to the firms in the largest private shareholder’s industry, to test whether information advantage is channel. As shown in Panal A in Table 1, the mean of *IndNPL* is 0.022. That is, the NPL ratio of the bank’s loans to the peers of the largest private shareholder is 2.2% on average. The mean of *FirmLoan* is 2.205, indicating that the maximum loan amount approved by banks to firms is 220.5 million CNY on average, with a maximum of 2 billion CNY. We use *Peer* to identify whether a firm receiving the loan is a peer of the largest private shareholder. As shown in Panel D of Table 1, the mean of *Peer* is 0.174, indicating that 17.4% of all borrowers of the bank on average are in the same industry as the largest private shareholder.

To demonstrate that the effect of the largest praivate shareholder on the industry choice of bank lending is unique, we also test the effect of the largest state-owned shareholder on bank lending. We measure the power of the largest state-owned shareholder by *LSS*, their ownership stake in the bank. Which excluding government shareholders, because we cannot identify the industry of government shareholders. We use *IndLoanS*, the ratio of bank loans to the industry of the largest state-owned shareholder to total loans, to measure the industry choice of bank lending. As shown in Panal A of Table 1, the mean of *IndLoanS* is 0.059. Banks’ loans to the industry of the largest state-owned shareholder accounted for 5.9% of the total annual loans on average, this is much smaller than 0.158 of *IndLoan*. The mean of *LSS* is 0.114, indicating that the largest state-owned shareholder owns 11.4% of the bank’s ownership stake on average, this is more than 0.078 of *LPS*. Interestingly, greater ownership stakes in banks by the largest state-owned shareholder do not lead banks to make more loans to their industries. As shown in S2 Appendix, *LPS* has a positive correlation with *IndLoan*, but *LSS* has a negative correlation.

Some of our tests used control variables. Characteristic variables at the bank level: *Size* is the logarithm of the bank’s total assets. *LDR* is the loan-to-deposit ratio. *Fore* is a dummy variable that equals 1 if the bank’s shares held by foreign investors. *CAR* is capital adequacy ratio. *Stat* is a dummy variable that equals 1 if the state-owned shareholder whose ownership stake is higher than the largest private shareholder of the bank is peer of the largest private shareholder. *Priv* is a dummy variable that equals 1 if the private shareholder whose ownership stake is higher than the largest state-owned shareholder of the bank is peer of the largest state-owned shareholder. Characteristic variables of regional macroeconomics: *GDPr* is the economic growth rate. *Deptr* is the deposit growth rate. *SOE* is the ratio of total state-owned assets to GDP. *GDP*_*sec* is the ratio of the GDP of the secondary industry to the total GDP. *GDP*_*thir* is the ratio of the GDP of tertiary industry to total GDP. Characteristic variables at the firm level: *LnAssets* is the logarithm of the firm’s total assets. *Leverage* is the sum of long-term debt and debt in current liabilities divided by total assets. *Current* is the current assets divided by total assets. *Profitability* is the net profit divided by total assets. *Capx* is the capital expenditure scaled by total assets.

On the basis of variable setting, in order to study the influence of the largest private shareholder on the industry choice of bank loans, the OLS regression equation with fixed effects is as follows.

$$\begin{array}{ccc} IndLoan_{it} & = & \beta_{0}+\beta_{1}LPS_{it}+\theta X+\omega_{it}+\eta_{t}+\epsilon_{it}, \end{array}$$ (1)

In Eq (1), *IndLoan*_*it*_ is the ratio of bank *i*’s loans to the industry of the largest private shareholder to the total loans in year *t*. *LPS*_*it*_ is Bank *i*’s largest private shareholder’s ownership stake in the bank in year *t*. *ω*_*it*_ is the industry fixed effect of the largest private shareholder. *η*_*t*_ is the year fixed effect. *ϵ*_*it*_ is is the error term. *X* are control variables, including characteristic variables at the bank level and regional macroeconomic characteristic variables.

In order to verify the information advantage of the largest private shareholder. On the one hand, we examine the change in the NPL ratio of the bank’s loans to the industry in which the largest private shareholder is located as the ownership stake of the largest private shareholder in the bank increases. The OLS regression equation with fixed effects is as follows.

$$\begin{array}{ccc} IndNPL_{it} & = & \beta_{0}+\beta_{1}LPS_{it}+\theta X+\omega_{it}+\eta_{t}+\epsilon_{it}, \end{array}$$ (2)

In Eq (2), *IndNPL*_*it*_ is the NPL ratio of bank *i*’s loans to the industry of the largest private shareholder in year *t*. Similar to Eq (1), *LPS*_*it*_ is Bank *i*’s largest private shareholder’s ownership stake in the bank in year *t*. *ω*_*it*_ is the industry fixed effect. *η*_*t*_ is the year fixed effect. *ϵ*_*it*_ is is the error term. *X* are control variables, including characteristic variables at the bank level and regional macroeconomic characteristic variables.

On the other hand, we examine whether the firms in the same industry have more loan amount at the bank as the largest private shareholder has a higher ownership stake in the bank. The OLS regression equation with fixed effects is as follows.

$$\begin{array}{ccc} FirmLoan_{ijt} & = & \beta_{0}+\beta_{1}LPS_{it}+\beta_{2}Peer_{ijt}\times LPS_{it}+\theta X^{{}^{\prime }}+\omega_{jt}+\eta_{t}+\epsilon_{it}, \end{array}$$ (3)

In Eq (3), *FirmLoan*_*ijt*_ is the maximum loan amount approved by bank *i* to firm *j* in year *t*. *LPS*_*it*_ is Bank *i*’s largest private shareholder’s ownership stake in the bank in year *t*. *Peer*_*ijt*_ is a dummy variable equals 1 if the firm *j* and the largest private shareholder of bank *i* belong to the same industry in year *t*. *β*_2_ represents the marginal effect of the largest private shareholder’s ownership stake in the bank on industry lending when the firm and the largest private shareholder of the bank are peers. Which is our key focus. *X*′ are characteristic variables at the firm level. *ω*_*jt*_ is the firm’s industry fixed effect, *η*_*t*_ is the year fixed effect and *ϵ*_*it*_ is is the error term.

## Results

### The impact of the largest private shareholder on bank lending to their industry

Combining Eq (1), we first run an OLS regression based on bank loans to the industry of the largest private shareholder and the largest private shareholder’s ownership stake in the bank. Table 2 reports the results. In column 1, we do not include any fixed effects. The effect of *LPS* is positive and significant. In column 2, we introduce industry fixed effect of the largest private shareholder to absorb industry-specific effects on lending behavior. The effect of *LPS* is still positive and significant. In column 3, which is our preferred specification, we further introduce year fixed effects to control for bank lending industry preference in particular year. Again, the effect of *LPS* is positive and significant. The point estimate in column 3 shows that a 1% increase in the ownership stake of the largest private shareholder implies a 0.272% increase in the proportion of bank loans to the industry in which the largest private shareholder is located. Here, we have proved **Hypothesis 1**.

**Table 2:** The largest private shareholder and bank loans. This table reports the estimation results from OLS regressions analyzing the impact of the largest private shareholder on bank lending to their industry, with a different combination of fixed effects in each column. Columns 1–3 uses *IndLoan* as the dependent variable. The results reporting the coefficients of the control variables are shown in S3 Appendix. Variables are defined in S1 Appendix. The sample period is from 2007 to 2020. In parentheses are t-statistics. ***, **, * indicate statistical significance at the 1%, 5%, and 10% level, respectively.

	(1) *IndLoan*	(2) *IndLoan*	(3) *IndLoan*
*LPS*	0.445***	0.272***	0.288***
	(6.09)	(4.45)	(4.69)
Industry fixed effects of LPS	No	Yes	Yes
Year fixed effects	No	No	Yes
Observations	1216	1216	1216
R-squared	0.137	0.466	0.474

The estimation results in Table 2 show that the proportion of bank loans to the industry where the largest private shareholder is located is positively correlated with the largest private shareholder’s ownership stake in the bank. But this is not enough to illustrate causality. We present cleaner estimates from channel tests in the sections below.

### Channel: Information advantage

We tested the information advantage about the industry of the largest private shareholder from two aspects.

#### The impact of the largest private shareholder on the industry’s NPL ratio

In this section, combining Eq (2), we run an OLS regression based on the bank’s NPL ratio to the industry where the largest private shareholder is located and the largest private shareholder’s ownership stake in the bank. Table 3 reports the results. In column 1, we do not include any fixed effects. The effect of *LPS* is negative and significant. In column 2, we introduce industry fixed effect of the largest private shareholder to absorb industry-specific effects on banks’ NPL ratios. The effect of *LPS* is still negative and significant. In column 3, which is our preferred specification, we further introduce year fixed effect to control for the inherent impact on bank’s NPL ratios in particular year. Again, the effect of *LPS* is negative and significant. The point estimate in column 3 shows that a 1% increase in the ownership stake of the largest private shareholder means a 0.17% reduction in the bank’s NPL ratio in the industry where the largest private shareholder is located.

**Table 3:** The largest private shareholder and the industry’s NPL ratio. This table reports the estimation results from OLS regressions analyzing the impact of the largest private shareholder on the bank’s NPL ratio in their industry, with a different combination of fixed effects in each column. Columns 1–3 uses *IndNPL* as the dependent variable. Variables are defined in S1 Appendix. The sample period is from 2007 to 2020. In parentheses are t-statistics. ***, **, * indicate statistical significance at the 1%, 5%, and 10% level, respectively.

	(1) *IndLoan*	(2) *IndLoan*	(3) *IndLoan*
*LPS*	-0.178**	-0.172**	-0.170**
	(-2.22)	(-2.08)	(-2.05)
Industry fixed effects of LPS	No	Yes	Yes
Year fixed effects	No	No	Yes
Observations	165	165	165
R-squared	0.0556	0.318	0.379

The results in Table 3 show that the bank’s NPL ratio in the industry where the largest private shareholder is located is negatively correlated with the largest private shareholder’s ownership stake in the bank. This is precisely because the information advantages about the industry of the largest private shareholders enable banks to better control risks during the loan process. In other industries, banks suffer from a more severe information disadvantage, so it is rational to lend to the industry where the largest private shareholder is located.

Further, we interact *IndNPL* with *LPS*, and divided the sample into two groups according to the level of *IndNPL* to test the influence of the largest private shareholder. Table 4 reports these results. In column 1, the coefficient of the interaction term is negative and highly significant. The samples in columns 2 and 3 are banks with lower and higher *IndNPL*, respectively. In column 2, the effect of *LPS* is significantly positive. In column 3, the effect is not significant. This suggests that banks will increase lending to the industry with the largest private shareholder due to lower *IndNPL*.

**Table 4:** The largest private shareholder, the industry’s NPL ratio and bank loans. This table reports the estimation results from OLS regressions analyzing the impact of the largest private shareholder on bank lending to their industry, with a different level of the bank’s NPL ratio in their industry in each column. Columns 1–3 uses *IndLoan* as the dependent variable. Variables are defined in S1 Appendix. The sample period is from 2007 to 2020. In parentheses are t-statistics. ***, **, * indicate statistical significance at the 1%, 5%, and 10% level, respectively.

	(1) *IndLoan*	(2) Lower *IndNPL IndLoan*	(3) Higher *IndNPL IndLoan*
*IndNPL* × *LPS*	-18.287***		
	(-4.25)		
*LPS*	0.712***	1.193***	0.417
	(-3.53)	(-2.87)	(-1.49)
Industry fixed effects of LPS	Yes	Yes	Yes
Year fixed effects	Yes	Yes	Yes
Observations	165	83	82
R-squared	0.621	0.457	0.598

#### The impact of the bank’s largest private shareholder on the bank’s loans to firms in the their industry

In this section, we use the loan data of listed firms. And combining Eq (3), we run an OLS regression based on the bank’s maximum loan amount to firms in the industry where the largest private shareholder is located and the largest private shareholder’s ownership stake in the bank. Table 5 reports the results. *Peer* is a dummy variable that equals 1 if the listed firm that obtains the bank’s loan and the bank’s largest private shareholder are in the same industry (with not peers replaced by 0). We interact *Peer* with *LPS*. We focus on the coefficient of the interaction term. In column 1, we do not include any fixed effects. The coefficient on the interaction term is positive and significant. In column 2, we introduce the individual fixed effects of firms receiving loans to absorb the specific effects of different individuals on loan demand. The coefficient on the interaction term is still positive and significant. In column 3, which is our preferred specification, we further introduce year fixed effects to control for changes in firm’s loan demand across years. Again, the coefficient on the interaction term is positive and significant. The point estimate in column 3 shows that a 1% increase in the ownership stake of the largest private shareholder in the bank means that firms in the same industry receive 7.448% more maximum loans amount from the bank than other firms. In column 4, we use the logarithm of one plus *FirmLoan* as the dependent variable. The coefficient on the interaction term remains highly significant and economically meaningful.

**Table 5:** The largest private shareholder and bank loans to firms in the their industry. This table reports the estimation results from OLS regressions analyzing the impact of the bank’s largest private shareholder on the bank’s loans to firms in the their industry, with a different combination of fixed effects in each column. Columns 1–3 uses *FirmLoan* as the dependent variable. Columns 4 uses *Ln*(1+ *FirmLoan*) as the dependent variable. Variables are defined in S1 Appendix. The sample period is from 2007 to 2020. In parentheses are t-statistics. ***, **, * indicate statistical significance at the 1%, 5%, and 10% level, respectively.

	(1) *FirmLoan*	(2) *FirmLoan*	(3) *FirmLoan*	(3) *Ln*(1+ *FirmLoan*)
*Peer* × *LPS*	7.448*	5.111**	5.163**	1.834***
	(1.82)	(1.98)	(1.98)	(2.74)
*LPS*	4.246	4.931**	5.358**	1.090**
	(1.35)	(2.31)	(2.44)	(2.16)
Firm fixed effects	No	Yes	Yes	Yes
Year fixed effects	No	No	Yes	Yes
Observations	735	735	735	735
R-squared	0.128	0.864	0.866	0.832

The results in Table 5 show that the bank’s maximum loan amount to firms in the industry where the largest private shareholder is located is positively correlated with the largest private shareholder’s ownership stake in the bank. This is also because the information advantage about the industry of the largest private shareholder makes the bank’s information collection cost for these peers lower. Compared with other industries, banks have lower costs to lend to peers of largest private shareholder, so banks made more loans to their peers. Here, we have proved **Hypothesis 2** by combining the estimation results in Tables 3, 4 and 5.

### Robustness

We consider a host of robustness tests to corroborate the documented effect of the largest private shareholder.

#### IV estimation

The bank’s preference for a certain industry makes the industry develop rapidly. This allows high-quality private enterprises in the industry to hold shares in banks after they grow. There is potential mutual causality between the largest private shareholder’s ownership stake of the bank and the industry choice of bank loans, resulting in endogeneity. We refer to the methods of existing research [20–22], and use *LPS*_*IV*, the average value of the largest private shareholder ownership stake of other banks located in the same region in the initial year of the bank sample (excluding their own situation), as an instrumental variable, and run 2SLS regression. The rationale for IV is that the ownership structure of an individual bank is related to its regional average, but the current industry choice of a bank’s lending does not affect the historical average ownership structure. Table 6 reports the results. Column 1 shows the first stage. *LPS*_*IV* has a positive and highly significant relationship with *LPS*. The first-stage F test reports a solid 12.801, the LM statistic for the over-identification test is 28.00, and the F statistic for the weak identification test is 27.84, indicating that *LPS*_*IV* is a valid instrument. Column 2 reports the second stage. The effect of *LPS* is positive and significant. The IV estimation has bolstered the credibility of the influence of the largest private shareholder on bank lending.

**Table 6:** The largest private shareholder and bank loans: IV estimation. This table reports the estimation results from 2SLS regressions analyzing impact of the largest private shareholder on bank lending to their industry, with industry fixed effect of the largest private shareholder and year fixed effect. Column 1 is the first stage regression of *LPS* on *LPS*_*IV*. Columns 2 is the second stage results. Columns 2 use *IndLoan* as the dependent variable. Variables are defined in S1 Appendix. The sample period is from 2007 to 2020. In parentheses are t-statistics. ***, **, * indicate statistical significance at the 1%, 5%, and 10% level, respectively.

	(1) *LPS*	(2) *IndLoan*
*LPS*_*IV*	0.225***	
	(5.28)	
*LPS*		1.163***
		(2.69)
Industry fixed effects of LPS	Yes	Yes
Year fixed effects	Yes	Yes
Observations	1216	1216
R-squared	0.269	0.379
First-stage F test	12.801	
LM statistic	28.00	
F statistic	27.84	

#### Alternative sample

A bank’s portfolio weight of loans in an industry may be high simply because that industry accounts for a large share in the overall economy. In these cases, it might not be surprising to see that the proportion of bank’s loans to that industry is relatively large as well. Although industry fixed effects of the largest private shareholder can absorb the type of heterogeneity to a large extent, we construct alternative sample measures to directly tackle the issue. Specifically, we removed the top three industry in each region with a share of the overall economy, which has ensured that the industry where the largest private shareholder is located is no longer the most important. Table 7 reports the results for the new sample. The effect of *LPS* remains highly significant and positive. The estimates still show that the impact of the largest private shareholder is true and accurate.

**Table 7:** The largest private shareholder and bank loans: Alternative sample. This table reports the estimation results from OLS regressions analyzing the impact of the largest private shareholder on bank lending to their industry, with a different combination of fixed effects in each column. Columns 1–3 uses *IndLoan* as the dependent variable. Variables are defined in S1 Appendix. The top three industry in each region with a share of the overall economy has been removed. The sample period is from 2007 to 2020. In parentheses are t-statistics. ***, **, * indicate statistical significance at the 1%, 5%, and 10% level, respectively.

	(1) *IndLoan*	(2) *IndLoan*	(3) *IndLoan*
*LPS*	0.366***	0.218***	0.232***
	(5.07)	(3.56)	(3.76)
Industry fixed effects of LPS	No	Yes	Yes
Year fixed effects	No	No	Yes
Observations	896	896	896
R-squared	0.155	0.505	0.514

#### Group test

Of course, the issue of endogeneity needs further discussion. For example, the large banks’ loans are more diversified. The loans of local small banks may have strong industry characteristics, and the loans will be inclined to certain specific industries. On the contrary, it is also easier for companies to take shares in local small banks, resulting in a high proportion of private large shareholders in local small banks. In other words, the amount of bank lending to the industry may be due to the nature of the bank itself and less relationship with the bank’s major shareholders.

To remove the distraction of this situation, we conduct a group test. We divide the sample into three groups according to the size of the total assets of banks in each year, namely small, medium and large banks. Table 8 reports the results. The effects of *LPS* in each column are positive and significant. This shows that the impact of the largest private shareholder is stable and reliable.

**Table 8:** The largest private shareholder and bank loans: Group test. This table reports the estimation results from OLS regressions analyzing the impact of the largest private shareholder on bank lending to their industry, with a different sample in each column. Columns 1–3 uses *IndLoan* as the dependent variable. Variables are defined in S1 Appendix. The top three industry in each region with a share of the overall economy has been removed. The sample period is from 2007 to 2020. In parentheses are t-statistics. ***, **, * indicate statistical significance at the 1%, 5%, and 10% level, respectively.

	(1) Small banks *IndLoan*	(2) Medium banks *IndLoan*	(3) Large banks *IndLoan*
*LPS*	0.238**	0.559***	0.253**
	(2.55)	(4.23)	(2.40)
Industry fixed effects of LPS	Yes	Yes	Yes
Year fixed effects	Yes	Yes	Yes
Observations	408	406	402
R-squared	0.494	0.521	0.505

#### The impact of the largest state-owned shareholder on bank lending to their industry

Our previous analysis has confirmed the influence of the largest private shareholder on the industry choice of bank lending. But we have not yet determined whether this effect is unique to the largest private shareholder. If it is not unique, we cannot get more research significance.

We test in this section whether the industry choice of bank lending is influenced by the largest state-owned shareholder. According to Eq (1), we replace the independent variable with *LSS*, the ownership stake of the largest state-owned shareholder in the bank, and replace the dependent variable with *IndLoanS*, the ratio of bank loans to the industry of the largest state-owned shareholder to total loans. we run an OLS regression based on bank loans to the industry of the largest state-owned shareholder and the largest state-owned shareholder’s ownership stake in the bank. Table 9 reports the results. In column 1, we do not include any fixed effects. The effect of *LSS* is positive but not significant. In column 2, we introduce industry fixed effects of the largest state-owned shareholder to absorb industry-specific effects on lending behavior. The effect of *LSS* is positive but still insignificant. In column 3, we further introduce a year fixed effect to control for bank loan industry preference at a particular time. Again, the effect of *LSS* is positive but not significant. The estimates suggest that the largest state-owned shareholders cannot influence banks to make a more loans to their industries. It also shows that the largest private shareholder has a different influence than the state-owned shareholder. We demonstrate that the influence of private large shareholders is unique.

**Table 9:** The largest state-owned shareholder and bank loans. This table reports the estimation results from OLS regressions analyzing the impact of the largest state-owned shareholder on bank lending to their industry, with a different combination of fixed effects in each column. Columns 1–3 uses *IndLoanS* as the dependent variable. Variables are defined in S1 Appendix. The sample period is from 2007 to 2020. In parentheses are t-statistics. ***, **, * indicate statistical significance at the 1%, 5%, and 10% level, respectively.

	(1) *IndLoan*	(2) *IndLoan*	(3) *IndLoan*
*LSS*	0.0306	0.0200	0.0223
	(1.58)	(1.29)	(1.44)
Industry fixed effects of LSS	No	Yes	Yes
Year fixed effects	No	No	Yes
Observations	1125	1125	1125
R-squared	0.0812	0.544	0.552

Therefore, our study has more practical significance. State-owned shareholders are ultimately owned by the government. They pay more attention to political interests, such as employment, social welfare and public goods, etc. They intervene in bank lending to satisfy the government’s preferences. This frees them from the consequences when the loan is used inefficiently. This results in information advantage not being critical. However, private shareholders are profit-seeking and must take the risk of bad loans. Consequently, the largest private shareholders must use their information advantage to ensure that their lending decisions are “right”.

### How the largest private shareholders influence bank’s loan: Manager or board

In this subsection, we mainly discuss how the largest private shareholder influences bank lending decisions. The management and the board of directors are important ways to influence the operation of the bank. The largest private shareholder can appoint directors and nominate executives. So are bank lending decisions made by major shareholders as a bank manager or by intervening in board decisions? We discuss this issue based on whether the bank’s chairman of the board and president are appointed by the largest private shareholder. Table 10 reports the results. *Chairman* is a dummy variable that equals 1 if the bank’s chairman of the board is appointed by the largest private shareholder. We interact *Chairman* with *LPS*. *President* is a dummy variable that equals 1 if the bank’s manager (CEO) is nominated by the largest private shareholder. We also interact *President* with *LPS*. We focus on the coefficients of the interaction terms.

**Table 10:** How the largest private shareholders influence bank’s loan: Manager or board. This table reports the estimation results from OLS regressions analyzing the impact of the largest private shareholder on bank lending to their industry, with a different interaction terms for each column. Columns 1–2 uses *IndLoan* as the dependent variable. Variables are defined in S1 Appendix. The sample period is from 2007 to 2020. In parentheses are t-statistics. ***, **, * indicate statistical significance at the 1%, 5%, and 10% level, respectively.

	(1) *IndLoan*	(2) *IndLoan*
*Chairman* × *LPS*	0.146**	
	(2.00)	
*President* × *LPS*		0.0347
		(0.50)
*LPS*	0.221***	0.279***
	(3.17)	(4.35)
Industry fixed effects of LPS	Yes	Yes
Year fixed effects	Yes	Yes
Observations	1216	1216
R-squared	0.475	0.474

In column 1, the coefficient on the interaction term is positive and significant. However, the coefficient is not significant in column 2. It shows that the largest private shareholder influences bank lending decisions by intervening in the board rather than the bank manager. The largest private shareholder has increased its influence on bank lending by appointing the chairman of the board.

### The role of ownership structure

We further analyze the role of ownership structure on the impact of the largest private shareholder in this subsection by adopting the concentration of ownership, the diversity of shareholders and the role of state-owned shareholders respectively. Table 11 reports the results. *Top*3 is the ownership shares of the top three shareholders (excluding the largest private shareholder). *HHI* is the Herfindahl-Hirschman index of ownership shares of the top ten shareholders. *LSS* is the ownership stake of the largest state-owned shareholder. We interact *Top*3, *HHI*, and *LSS* with *LPS*, respectively. We focus on the coefficients of the interaction terms.

**Table 11:** The role of ownership structure. This table reports the estimation results from OLS regressions analyzing the impact of the largest private shareholder on bank lending to their industry taking into account the role of ownership structure. Columns 1–3 uses *IndLoan* as the dependent variable. Variables are defined in S1 Appendix. The sample period is from 2007 to 2020. In parentheses are t-statistics. ***, **, * indicate statistical significance at the 1%, 5%, and 10% level, respectively.

	(1) *IndLoan*	(2) *IndLoan*	(3) *IndLoan*
*Top*3 × *LPS*	-0.973***		
	(-4.55)		
*HHI* × *LPS*		-2.596***	
		(-4.58)	
*LSS* × *LPS*			-2.031***
			(-5.31)
*LPS*	0.584***	0.619***	0.388***
	(6.55)	(6.55)	(6.10)
Industry fixed effects of LPS	Yes	Yes	Yes
Year fixed effects	Yes	Yes	Yes
Observations	1216	1216	1216
R-squared	0.483	0.483	0.486

In column 1, the coefficient on the interaction term is negative and significant. It shows that the more concentrated the ownership shares of the top three shareholders, the more they can restrain the influence of the largest private shareholder on bank loans. In column2, the coefficient on the interaction term is also negative and significant. The smaller the *HHI*, the more dispersed the ownership of shareholders (the higher the diversity of shareholders). They cannot form constraints on the largest private shareholder, so this magnifies the influence of the largest private shareholder. In column 3, the coefficient on the interaction term is again negative and significant. This is very interesting. It shows that although the largest state-owned shareholders cannot facilitate banks’ lending to their own industries, they can limit the influence of the largest private shareholders.

## Conclusion

We contribute to an in-depth understanding of the role of bank ownership structure in the allocation of bank loans. Our study is an earlier research based on the perspectives of shareholders’ industries and banks’ industry lending. Our findings suggest that the largest private shareholder has an information advantage in their industry, which enables banks to reduce the cost of collecting information on the industry and alleviate information asymmetry, which helps banks to provide more loans to the industry.

The industry choice of bank loans determines the economic structure and industrial structure to a certain extent. Our study has important implications for a developing economy like China. The governments of these countries often intervene in bank lending behavior, forcing bank loans to match national economic development goals. We find that the industry background of the largest private shareholder and the ownership stake in the bank can have a significant impact on the industry choice of bank loans. Therefore, guiding financial resources to support the adjustment and upgrading of the economic structure can be achieved by introducing relevant high-quality private enterprises. For regulators, the introduction of future regulatory policies should consider the influence of the largest private shareholder. Supervisors need to focus on private shareholders’ ownership stakes in banks and carefully assess their ability to take on bank risks.

Our research provides insight into how the largest private shareholder affects bank lending behavior. We enrich the theory of corporate governance and information asymmetry. For future research, we can focus on how banks limit the tunneling of large shareholders. In addition, whether excessively high proportion of industry loans generate higher risks and how does the bank control are also some directions.

## Supporting information

S1 Appendix (PDF)

S2 Appendix (PDF)

S3 Appendix (PDF)

S1 Data (XLSX)
