# Supplementary material for: The influence of the largest private shareholder on bank loans: Evidence from China
Source: PLoS One. 2022 Oct 27;17(10):e0276877. doi: 10.1371/journal.pone.0276877 (PMC9612559; doi:10.1371/journal.pone.0276877)
Supplement: S1 Appendix — (PDF) [file pone.0276877.s001.pdf]

Appendix

Table A1. Variable definitions.

Variable	Definition
IndLoan	The ratio of bank's loans on the industry in which the largest private shareholder is located to bank's total loans.
LPS	The largest private shareholder's ownership stake in the bank.
IndNPL	The NPL ratio of bank loans to the industry in which the largest private shareholder is located.
FirmLoan (100 mln CNY)	The maximum loan amount approved by the bank to a firm in the industry in which the largest private shareholder is located.
$Ln(1 + FirmLoan)$	The natural logarithm of 1+*FirmLoan*.
IndLoanS	The ratio of bank's loans on the industry in which the largest state-owned shareholder is located to bank's total loans.
LSS	The largest state-owned shareholder's ownership stake in the bank. Which excluding government shareholders, because we cannot identify the industry of government shareholders.
Stat	A dummy variable that equals 1 if the state-owned shareholder whose ownership stake is higher than the largest private shareholder of the bank is peer (in the same industry) of the largest private shareholder.
Priv	A dummy variable that equals 1 if the private shareholder whose ownership stake is higher than the largest state-owned shareholder of the bank is peer (in the same industry) of the largest state-owned shareholder.
Size (CNY)	The natural logarithm of the bank's total assets.
LDR	The loan-to-deposit ratio.
Fore	A dummy variable that equals 1 if the bank's shares held by foreign investors.
CAR	The capital adequacy ratio.
Chairman	A dummy variable that equals 1 if the bank's chairman of the board is appointed by the largest private shareholder.
President	A dummy variable that equals 1 if the bank's manager (CEO) is nominated by the largest private shareholder.
*Top*3	The ownership shares of the top three shareholders (excluding the largest private shareholder).
HHI	The Herfindahl-Hirschman index of ownership shares of the top ten shareholders.
GDPr	The economic growth rate.
Deptr	The deposit growth rate.
SOE	The ratio of total state-owned assets to GDP.
GDP_sec	The ratio of the GDP of the secondary industry to the total GDP.
GDP_thir	The ratio of the GDP of tertiary industry to total GDP.
Peer	A dummy variable that equals 1 if the firm that have obtained loans from this bank and the largest private shareholder of bank belong to the same industry.
LnAssets (CNY)	The natural logarithm of the firm's total assets.
Leverage	The sum of long-term debt and debt in current liabilities divided by the firm's total assets.
Current	The current assets divided by the firm's total assets.
Profitability	The net profit divided by the firm's total assets.
Capx	The capital expenditure scaled by the firm's total assets.
