# Supplementary material for: The influence of the largest private shareholder on bank loans: Evidence from China
Source: PLoS One. 2022 Oct 27;17(10):e0276877. doi: 10.1371/journal.pone.0276877 (PMC9612559; doi:10.1371/journal.pone.0276877)
Supplement: S2 Appendix — (PDF) [file pone.0276877.s002.pdf]

Appendix

Table A2. Correlation Matrix

This table reports the correlation matrix. Variables are defined in Appendix Table A1. The sample period is from 2007 to 2020.

	1	*2*	*3*	*4*	*5*	*6*	*7*
1 IndLoan	1						
2 LPS	0.247***	1					
3 LSS	-0.182***	-0.241***	1				
4 Size	-0.218***	-0.266***	-0.025	1			
5 LDR	0.080***	-0.025	-0.109***	0.189***	1		
6 Fore	-0.054*	-0.122***	-0.109***	0.353***	0.109***	1	
7 CAR	-0.001	0.021	0.073**	-0.211***	-0.016	-0.056*	1
8 Stat	-0.087***	-0.124***	0.097***	0.057**	-0.049*	0.036	0.069**
9 GDPr	-0.017	-0.134***	0.028	-0.349***	-0.227***	-0.008	0.081***
10 Deptr	0.008	-0.127***	0.015	-0.287***	-0.207***	0.005	0.025
11 SOE	-0.134***	-0.072**	0.047*	0.267***	0.151***	0.035	-0.001
12 GDP_sec	0.126***	0.017	-0.026	-0.459***	-0.217***	-0.053*	0.028
13 GDP_thir	-0.041	-0.014	-0.008	0.530***	0.348***	0.168***	-0.063**

	8	*9*	*10*	*11*	*12*	*13*	
1 IndLoan							
2 LPS							
3 LSS							
4 Size							
5 LDR							
6 Fore							
7 CAR							
8 Stat	1						
9 GDPr	0.087***	1					
10 Deptr	-0.011	0.577***	1				
11 SOE	0.070**	-0.292***	-0.115***	1			
12 GDP_sec	-0.095***	0.589***	0.467***	-0.361***	1		
13 GDP_thir	0.028	-0.606***	-0.466***	0.351***	-0.862***	1	
