# Supplementary material for: The influence of the largest private shareholder on bank loans: Evidence from China
Source: PLoS One. 2022 Oct 27;17(10):e0276877. doi: 10.1371/journal.pone.0276877 (PMC9612559; doi:10.1371/journal.pone.0276877)
Supplement: S3 Appendix — (PDF) [file pone.0276877.s003.pdf]

Appendix

Table A3. The largest private shareholder and bank loans.

	(1) *IndLoan*	(2) *IndLoan*	(3) *IndLoan*
LPS	0.445***	0.272***	0.288***
	(6.09)	(4.45)	(4.69)
Stat	-0.00789	0.00636	0.00578
	(-0.45)	(0.42)	(0.38)
Size	-0.0248***	-0.0179***	-0.0160***
	(-5.70)	(-5.02)	(-4.23)
LDR	0.0887***	0.134***	0.153***
	(2.93)	(5.51)	(6.05)
Fore	-0.000280	0.00872	0.00639
	(-0.03)	(1.27)	(0.91)
CAR	-0.107	-0.0969	-0.105
	(-1.06)	(-1.19)	(-1.28)
GDPr	-0.341**	-0.318**	-0.567***
	(-2.17)	(-2.53)	(-3.08)
Deptr	0.0483	0.0234	-0.00275
	(0.63)	(0.38)	(-0.03)
SOE	-0.0384***	-0.0239**	-0.0223*
	(-3.15)	(-2.45)	(-1.67)
GDP_sec	0.595***	0.344***	0.273***
	(5.45)	(3.87)	(2.98)
GDP_thir	0.504***	0.226***	0.206**
	(4.92)	(2.64)	(2.36)
Industry fixed effects of LPS	No	Yes	Yes
Year fixed effects	No	No	Yes
Observations	1216	1216	1216
R-squared	0.137	0.466	0.474
